# Clinical factors associated with need for neurosurgical care in young children with imaging for macrocephaly: a case control study

**DOI:** 10.1186/s12887-023-04379-2

**Published:** 2023-11-04

**Authors:** Jessica F. Rohde, Jeffrey Campbell, Julie Barbera, Elena Taylor, Ashok Ramachandra, Christopher Gegg, Andrea Scherer, Joseph Piatt

**Affiliations:** 1 Division of General Pediatrics, Nemours Children’s Hospital, 1600 Rockland Road, Wilmington, DE 19803 USA; 2 Department of Pediatrics, Sidney Kimmel Medical College, Thomas Jefferson University, Philadelphia, PA USA; 3 Division of Neurosurgery, Nemours Children’s Hospital, Wilmington, DE USA; 4 Department of Neurological Surgery, Sidney Kimmel Medical College, Thomas Jefferson University, Philadelphia, PA USA; 5 Sidney Kimmel Medical College, Thomas Jefferson University, Philadelphia, PA USA; 6 Department of Radiology, Nemours Children’s Hospital, Wilmington, DE USA; 7 Division of Neurosurgery, Nemours Children’s Hospital, Orlando, FL USA

**Keywords:** Imaging, Macrocephaly, Pediatrics, Primary care, Risk factors, Neurosurgery

## Abstract

**Background:**

Macrocephaly is present in 2.3% of children with important neurosurgical conditions in the differential diagnosis. The objective of this study was to identify clinical associations with actionable imaging findings among children with head imaging for macrocephaly.

**Methods:**

We conducted a case-control study of head imaging studies ordered for macrocephaly among children 24 months and younger in a multistate children’s health system. Four neurosurgeons reviewed the images, determining cases to be a ‘concern’ if neurosurgical follow-up or intervention was indicated. Electronic health records were reviewed to collect patient-level data and to determine if surgery was performed. Controls were matched 3:1 to cases of ‘concern’ in a multivariate model using conditional logistic regression.

**Results:**

In the study sample (*n* = 1293), 46 (4%) were concern cases, with 15 (1%) requiring surgery. Significant clinical factors associated with neurosurgical concern were bulging fontanel [aOR 7.47, (95% CI: 2.28–24.44), *P* < 0.001], prematurity [aOR 21.26, (95% CI: 3.76–120.21), *P* < 0.001], any delay [aOR 2.67, (95% CI: 1.13–6.27), *P* = 0.03], and head-weight Z-score difference (W_diff, defined as the difference between the Z-scores of head circumference and weight) [aOR 1.70, (95% CI: 1.22–2.37), *P* = 0.002].

**Conclusions:**

Head imaging for macrocephaly identified few patients with findings of concern and fewer requiring surgery. A greater head-weight Z-score difference appears to represent a novel risk factor for neurosurgical follow-up or intervention.

## Background

Macrocephaly in children is defined as a head circumference greater than 2 standard deviations above the mean, or above the 97.7th percentile on the World Health Organization (WHO) growth chart [1]. Macrocephaly is present in 2.3% of children and is a non-specific finding with an important neurosurgical differential diagnosis; therefore, it is a common reason for referral to pediatric neurosurgeons [2]. Brain imaging is often ordered to determine the etiology of the increased head circumference [3]. Imaging in young children can be challenging because of simultaneous goals of limiting radiation exposure, reducing need for sedation, and managing expenses [3], as well as managing parental concerns related to imaging. Additionally, imaging is often low yield for neurosurgical conditions; a recent analysis of 538 imaging studies ordered for macrocephaly found only 7 cases of hydrocephalus and 1 chronic subdural hematoma [2].

Despite macrocephaly presenting a common and possibly concerning sign for a neurosurgical condition, there are currently no evidence-based guidelines for pediatric primary care providers to reference. Previous studies have aimed to identify risk factors that may be associated with important findings on imaging in macrocephalic children with varying results, including developmental delay [4, 5] and specific head circumference growth patterns, such as above the 95th, 97th, or 99.6th percentile or crossing multiple major percentile lines [6]. Our study aims to add to the limited existing evidence related to macrocephaly, risk factors, and indications for imaging by seeking to identify clinical associations with actionable imaging findings among children younger than 24 months of age who have macrocephaly.

## Methods

**Fig. 1:**
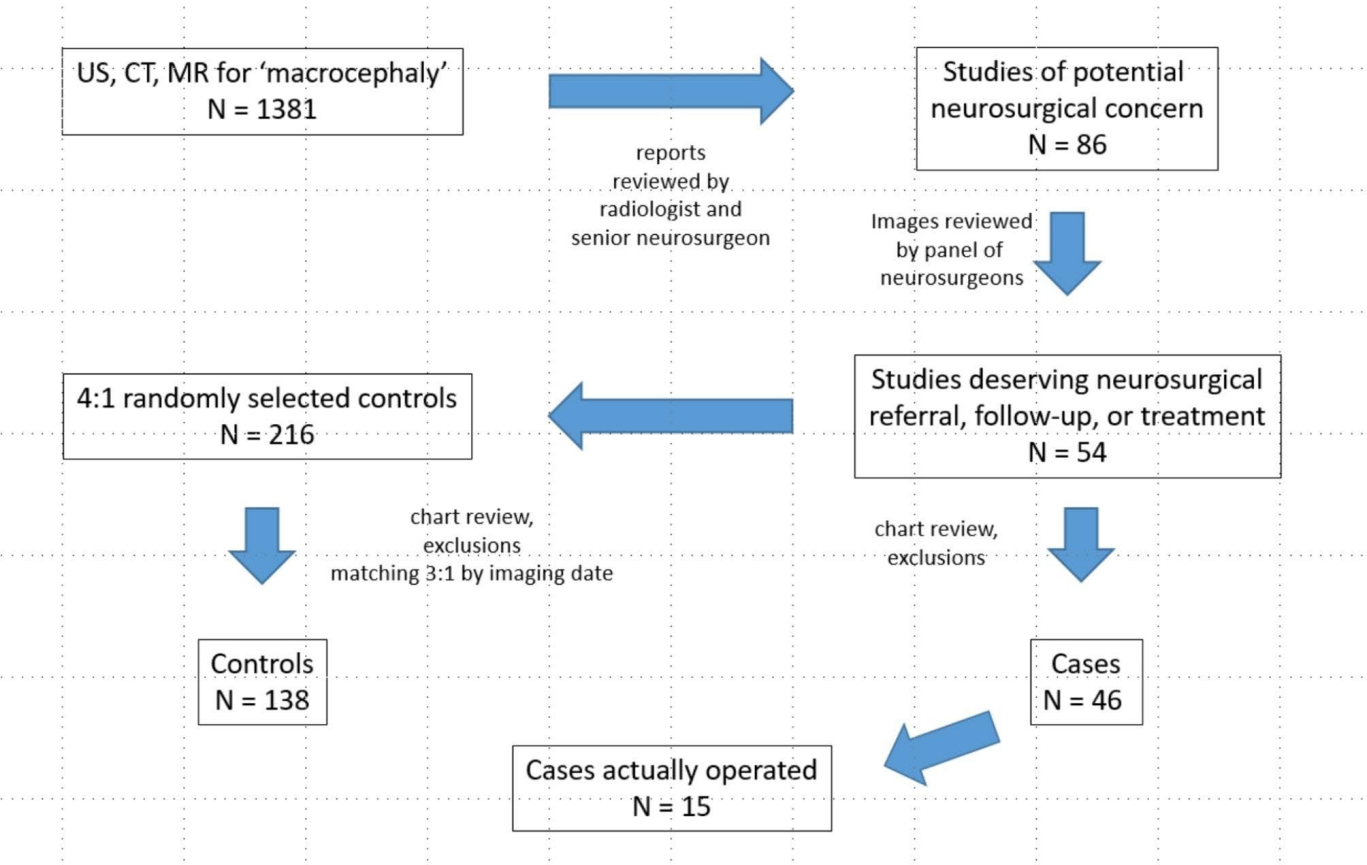
Flow chart of study sample distillation

A case-control study was undertaken using electronic records at 2 children’s hospitals (one in Delaware and one in Florida) and approved by the appropriate Institutional Review Board, reference #1572830, with a waiver for obtaining informed consent based on the applicable federal regulation. A radiology registry was queried by text search for head ultrasound (US), magnetic resonance (MR) imaging, or computed tomographic (CT) scanning studies with ‘macrocephaly,’ ‘macrocephalia,’ or ‘head growth’ in the requisition among children in the first 2 years of life during the period October 2011 to February 2020. Duplicate studies were expunged, and, if patients had more than one study, only the earliest was retained for analysis. A total of 1293 unique patients were identified. After review of the radiology reports by a radiologist and senior neurosurgeon,86 interpretations mentioned an abnormal finding (Fig. 1; Table 1). Findings noted on imaging studies of potential neurosurgical concern are listed in Table 2.

**Table 1:** Imaging Modalities Used. The imaging modalities used corresponding to potential, control, concern, and surgical cases as depicted in Fig. 1

Modality	All Studies (*n* = 1293)	Potential Cases (*n* = 86)	Control Cases (*n* = 138)	Concern Cases (*n* = 46)	Surgical Cases (*n* = 15)
CT	131 (10.1%)	11 (12.8%)	11 (8.0%)	2 (4.4%)	1 (6.7%)
MR	351 (27.1%)	36 (41.9%)	44 (31.9%)	16 (34.8%)	4 (26.7%)
US	811 (62.7%)	39 (45.3%)	83 (60.9%)	28 (60.9%)	10 (66.7%)

*CT* Computed tomography, *MR* Magnetic resonance imaging, *US* Ultrasound

**Table 2:** Findings on Imaging Studies Ordered for Macrocephaly. ﻿Many studies had more than one finding (n = 86)

Finding	Count
Ventriculomegaly	48
Enlargement of subarachnoid spaces	42
Subdural hygroma / hematoma	24
Developmental anomaly	16
Cyst	15
Cerebellar ectopia/Chiari	10
Intracerebral/intraventricular hemorrhage	6
Skull abnormalities	5
Tumor	4
Calcification	3
White matter abnormality	3
Vein of Galen aneurysm	2
Enlarged perivascular spaces	1

For all studies with interpretations mentioning an abnormality, four fellowship-trained pediatric neurosurgeons in active clinical practice independently reviewed the imaging. If any one of the neurosurgeons judged that the case merited neurosurgical referral, follow-up, or intervention, that case was considered a ‘concern.’ Benign enlargement of the subarachnoid spaces was not included among the cases of neurosurgical concern. Control cases were selected randomly from the remaining imaging studies in a 4:1 ratio.

Two investigators (not neurosurgeons) independently reviewed the electronic health records (EHR) of all concern and control cases. Study data were collected and managed using REDCap (Research Electronic Data Capture), a secure, web-based software platform designed to support data capture for research studies [7, 8]. These investigators reviewed all pertinent documents in the record up to the date of the imaging study and no later with the intention of blinding them to the study outcomes. Raw measurements and Z-scores from the WHO growth charts for head circumference, length, and weight at the date nearest the date of the imaging study were captured, with Z-scores adjusted for prematurity when gestational age was less than 36 weeks. While percentiles for growth parameters such as head circumference, length, and weight are more commonly used in primary care pediatrics, the Z-score represents the number of standard deviations away from the mean and is commonly used in other literature related to macrocephaly [2, 6, 9]. The senior author (a neurosurgeon) resolved discrepancies between the observations of the two investigators by further EHR review.

Exclusion criteria included diagnosis of any condition identified that might have been an indication for brain imaging apart from macrocephaly, such as focal neurological abnormalities; seizures; nystagmus; previous traumatic brain injury; previous neurosurgical intervention; central nervous system infection; no head circumference measurement in the chart; or a diagnosis of perinatal intraventricular hemorrhage. Prematurity and developmental delay were not exclusion criteria. It was not recorded as part of the chart review whether the patient had a neurosurgical consult. Missing data were handled conservatively. Missing data among categorical covariates were considered negative. Among quantitative covariates, the sample median was substituted.

Sufficient control cases (head imaging results that did not include abnormalities) remained for 3:1 matching with concern cases after exclusions. An iterative nearest-neighbor algorithm was utilized based on the date of the imaging study. Univariate associations with neurosurgical concern were assessed by cross-tabulation for categorical covariates and by the non-parametric Wilcoxon test for quantitative covariates, as none of them was normally distributed. Based on univariate associations significant at the *P* < 0.05 level, a multivariate model of neurosurgical concern was developed using manual stepwise backward conditional logistic regression. All *P*-values were 2-tailed.

A parallel secondary analysis was performed for the small subset of concern cases that came to neurosurgical intervention. Because of small numbers, scores were developed from a logistic regression model without matching. Surgical cases were compared with the rest of the study sample. Data were organized and analyzed in RStudio (R Foundation for Statistical Computing, Vienna, Austria).

## Results

In the study sample of children with macrocephaly who had head imaging before 24 months of age (*n* = 1293), 46 (4%) were concern cases with 138 matched controls (Table 3). There were 55 females (30%) in the entire study group of cases and matched controls (*n* = 184). The median age was 6.4 months (interquartile range 4.3 to 11.3 months). The median gestational age was 38 weeks (interquartile range 37 to 39 weeks).

**Table 3:** Demographic Data. Medians are shown with interquartile ranges in brackets

	Concern Cases *n* = 46	Control Cases *n* = 138	*P*-value
Age (months)	6.1 [4.3–11.8]	6.4 [4.3–10.8]	< 0.001
Sex			0.40
Female	11 (24%)	44 (32%)	
Male	35 (76%)	94 (68%)	
Head circumference Z-score	3.08 [2.41–4.52]	2.58 [2.12–3.16]	< 0.001
Weight Z-score	0.87 [-0.15–1.37]	0.55 [-0.34–1.45]	< 0.001
Length Z-score	0.53 [-0.53–1.21]	0.03 [-1.05–0.91]	0.08
Gestational age (weeks)	37 [36–39]	38 [37–39]	< 0.001

A case was considered a neurosurgical concern if any one of the four neurosurgical reviewers voted affirmatively. Of 46 concern cases, 23 (50%) received 4 votes, 6 (13%) received 3 votes, 10 (22%) received 2 votes, and 7 (15%) received only 1 vote. Among the neurosurgeons, the rate of affirmative votes ranged from 65 to 89%. Pair-wise Kappa values ranged from 0.38 to 0.55. Diagnoses among the concern cases included chronic subdural hematoma or hygroma, possible hydrocephalus, Chiari malformations, and possible benign brain tumors.

### Concern cases

The following factors were associated with neurosurgical concern on a univariate basis: head circumference Z-score (HC_Z); head-weight Z-score difference (W_diff, defined as the difference between the Z-scores of head circumference and weight); head-length Z-score difference (L_diff, defined as the difference between the Z-scores of head circumference and length); prematurity; any developmental delay; rapid acceleration of head growth (increase of greater than 2 standard deviations under observation); irritability; and, bulging of the anterior fontanel. The following factors were not associated with neurosurgical concern: actual macrocephaly (HC_Z greater than 2); age; delivery (vaginal or cesarean); feeding disturbance; sex; parental macrocephaly (very sparse data); skull deformity; sleep disturbance; or year of imaging study.

Stepwise backward conditional logistic regression yielded a model with the following significant covariates: bulging fontanel [aOR 7.47, (95% CI: 2.28–24.44), *P* < 0.001], prematurity [aOR 21.27, (95% CI: 3.76–120.21), *P* < 0.001], any delay [aOR 2.67, (95% CI: 1.13–6.27), *P* = 0.03], and W_diff [aOR 1.70, (95% CI: 1.22–2.37), *P* = 0.002] (Table 4). This model was analyzed with definitions of prematurity ranging from 28 weeks to 40 weeks. Based on the Akaike information criterion, gestational age younger than 34 weeks fit the data best; therefore, prematurity in the model was defined as gestational age younger than 34 weeks.

**Table 4:** Associations of covariates with neurosurgical concern. Associations of bulging fontanel, prematurity, any developmental delay, and ‘W_diff’ (difference between the Z-scores of head circumference and weight) with neurosurgical concern by conditional logistic regression

Covariate	Adjusted odds ratio	95% Confidence interval	*P*-value
Bulging fontanel	7.47	2.28–24.44	< 0.001
Any developmental delay	2.67	1.13–6.27	0.03
Prematurity	21.26	3.76–120.21	< 0.001
W_diff	1.70	1.22–2.37	0.002

### Surgical cases

There were only 15 surgical cases. Diagnoses included hydrocephalus (*n =* 9), subdural hygroma or hematoma (*n =* 3), brain tumor (*n =* 1), brain tumor with hydrocephalus (*n =* 1), and arachnoid cyst (*n =* 1). Cases came to surgery between 1 and 480 days after the imaging study (median 2 days, interquartile range 1 to 11 days). Eight of the 15 patients (53%) were admitted for surgery through the emergency department. No operations were performed at night. The following factors were associated with surgical intervention on a univariate basis: bulging fontanel, irritability, sleep disturbance, age, HC_Z, W_diff, and L_diff. Factors not associated with surgical intervention included macrocephaly based on Z-score; rapid acceleration of head circumference; skull deformity; mode of delivery; feeding disturbance; parental macrocephaly; prematurity; any developmental delay; or sex.

Stepwise backward logistic regression yielded a model with the following significant covariates: bulging fontanel [aOR 32.43, (95% CI: 5.67–185.60), *P* < 0.001], sleep disturbance [aOR 13.98, (95% CI: 2.20–88.71), *P* = 0.005], and W_diff [aOR 3.19, (95% CI: 1.70–6.00), *P* < 0.001] (Table 5).

**Table 5:** Associations of covariates with neurosurgical intervention. Associations of bulging fontanel, prematurity, any developmental delay, and ‘W_diff’ (difference between the Z-scores of head circumference and weight) with neurosurgical concern by conditional logistic regression

Covariate	Adjusted odds ratio	95% Confidence interval	*P*-value
bulging fontanel	32.43	5.67–185.60	< 0.001
sleep disturbance	13.98	2.20–88.71	0.005
W_diff	3.19	1.70–6.00	< 0.001

## Discussion

Given the limited evidence that exists for evaluation and management of macrocephaly in young children, we sought to identify clinical factors associated with head imaging studies requiring follow-up and surgical management. Of head imaging studies done, there were few patients with findings of concern (46/1293, 3.6%) and even fewer (15/1293, 1.2%) requiring surgery. Furthermore, of those identified as requiring neurosurgery follow-up, less than one-third ultimately required neurosurgery. These findings are similar to prior literature, although our rate of abnormal findings from imaging done for macrocephaly is lower than previously reported [4, 5].

Our findings indicated bulging of the fontanel and W_diff were associated with abnormal head imaging results requiring further neurosurgical follow-up and surgery. Given our sample of infants imaged for macrocephaly, the discrepancy for W_diff most likely reflects a larger head circumference Z-score compared with a smaller weight Z-score. The possibility that a discrepancy between head growth and somatic growth might be associated with intracranial pathology was suggested in a study of autism [10]. As an indicator for imaging of infants with macrocephaly, it seems to be novel. In the context of the current study sample of infants with macrocephaly, each additional point greater for W_diff is associated with an increased likelihood of imaging findings requiring neurosurgical follow-up or intervention. This finding supports the clinical gestalt that macrocephaly associated with a comparable degree of macrosomia tends to be benign.

That length Z-score dropped out of the model in preference to weight Z-score is curious. Among our control cases, we noted that head circumference Z-score was much more tightly correlated with weight Z-score than with length Z-score (data not shown). Accordingly, a condition that systematically affects head size can be expected to perturb its linkage with weight before its linkage with length.

### Clinical implications

For general pediatricians who commonly encounter infants and children with macrocephaly during routine primary care visits, it is helpful to know that few children undergoing head imaging for macrocephaly needed neurosurgery follow-up. Additionally, this investigation identified risk factors, including developmental delay, prematurity (gestational age less than 34 weeks), a bulging fontanel, or a larger W_diff, which may lead pediatricians to consider imaging for young children with macrocephaly. While further studies will need to validate a greater W_diff as a risk factor and investigate whether a critical threshold for W_diff exists, pediatricians may consider comparing the Z-scores for the head circumference and weight percentiles when assessing children with macrocephaly. Use of Z-scores is aided by the ability of EHR growth charts to display the Z-score along with the percentile for growth parameters, although a WHO head circumference growth chart by Z-score also exists [11].

For pediatric neurosurgeons, the finding of a larger W_diff as a significant risk factor for head imaging findings requiring neurosurgical follow-up supports the impression shared by neurosurgeons and seasoned pediatricians that a large head in relation to a larger body size is less likely to be concerning. As pediatric neurosurgeons undoubtedly receive many consults for macrocephaly, educating primary care pediatricians about risk factors to consider prior to referral or imaging may help limit unnecessary referrals and imaging, as well as stress to the family.

### Limitations

This case-control study based on chart review data has structural limitations to acknowledge. As for most institutional studies, there are questions of internal and external validity. Furthermore, a case-control study design is considered problematic for establishing diagnostic accuracy and could not reliably be used to generate decision rules to support imaging guidelines or an actionable threshold for W_diff [12].

Missing data were troublesome. Unfortunately, since a substantial fraction of patients were referred for imaging from primary practices not linked to our EHR, historical growth data for these patients were not available. Likewise, the clinical authors strongly suspect that parental macrocephaly is a predictor of benign infantile macrocephaly with prior literature supporting this hypothesis as well [13]. However, parental head circumference data were much too sparse in our EHR for analysis. If a prospective study is undertaken in the future, every effort must be made to obtain historical growth data and parental measurements.

The study outcome itself proved more problematic than anticipated. Review of imaging studies by four neurosurgeons was intended to enhance the generalizability of the outcome; however, it instead revealed an unanticipated degree of neurosurgical practice variation. More critically, neurosurgical ‘concern’ was chosen as a convenience outcome because there were too few cases requiring surgical intervention for robust analysis. To be identified as requiring follow-up is of limited value to a patient if no intervention is ever required.

## Conclusions

Macrocephaly in infants and young children is an issue commonly encountered by pediatricians without guidelines available to direct management. Our sample showed a very small percentage of children younger than age 2 years with imaging for macrocephaly had issues requiring neurosurgical follow-up, with even fewer requiring neurosurgical intervention. This paper provides support for identification of risk factors requiring imaging in the setting of a macrocephaly diagnosis, specifically developmental delay, prematurity (gestational age less than 34 weeks), a bulging fontanel, or a larger W_diff. Future directions for this work include a prospective study to replicate the importance of these variables and to assess their accuracy in predicting children at risk for needing neurosurgical follow-up or intervention.

## Data Availability

The datasets used and/or analyzed during the current study are available from the corresponding author on reasonable request.

## Abbreviations

aOR: adjusted odds ratio
CI: confidence interval
CT: computed tomography
EHR: electronic health record
HC_Z: head circumference Z-score
L_diff: difference between the Z-scores of head circumference and length
MR: magnetic resonance
US: ultrasound
W_diff: difference between the Z-scores of head circumference and weight
WHO: World Health Organization
